# Inter-relationships of risk factors and pathways associated with all-cause mortality in patients with chronic schizophrenia

**DOI:** 10.3389/fpsyt.2023.1309822

**Published:** 2024-05-20

**Authors:** Teng-Hung Yu, Thung-Lip Lee, Chin-Feng Hsuan, Cheng-Ching Wu, Chao-Ping Wang, Yung-Chuan Lu, Ching-Ting Wei, Fu-Mei Chung, Yau-Jiunn Lee, I-Ting Tsai, Wei-Hua Tang

**Affiliations:** ^1^Division of Cardiology, Department of Internal Medicine, E-Da Hospital, I-Shou University, Kaohsiung City, Taiwan; ^2^School of Medicine, College of Medicine, I-Shou University, Kaohsiung City, Taiwan; ^3^School of Medicine for International Students, College of Medicine, I-Shou University, Kaohsiung City, Taiwan; ^4^Division of Cardiology, Department of Internal Medicine, E-Da Dachang Hospital, I-Shou University, Kaohsiung City, Taiwan; ^5^Division of Cardiology, Department of Internal Medicine, E-Da Cancer Hospital, I-Shou University, Kaohsiung City, Taiwan; ^6^Division of Endocrinology and Metabolism, Department of Internal Medicine, E-Da Hospital, I-Shou University, Kaohsiung City, Taiwan; ^7^Division of General Surgery, Department of Surgery, E-Da Hospital, I-Shou University, Kaohsiung City, Taiwan; ^8^The School of Chinese Medicine for Post Baccalaureate, College of Medicine, I-Shou University, Kaohsiung City, Taiwan; ^9^Lee's Endocrinologic Clinic, Pingtung, Taiwan; ^10^Department of Emergency, E-Da Hospital, I-Shou University, Kaohsiung City, Taiwan; ^11^Division of Cardiology, Department of Internal Medicine, Taipei Veterans General Hospital, Yuli Branch, Hualien, Taiwan; ^12^Faculty of Medicine, School of Medicine, National Yang Ming Chiao Tung University, Taipei, Taiwan

**Keywords:** chronic schizophrenia, all-cause mortality, clinical and biochemical factors, lifestyle, comorbid illnesses, causal pathways, inter-relationship, Structural Equation Modeling

## Abstract

**Introduction:**

Of all psychiatric disorders, schizophrenia is associated with the highest risk of all-cause mortality. This study aimed to investigate independent risk factors for all-cause mortality in patients with chronic schizophrenia. In addition, the possible causal inter-relationships among these independent risk factors and all-cause mortality were also explored.

**Methods:**

We conducted an analysis of 1,126 patients with chronic schizophrenia from our psychiatric department from April 2003 to August 2022, and retrospectively reviewed their medical records. The study endpoint was all-cause mortality. Baseline clinical characteristics including sociodemographic data, biochemical data, lifestyle factors, comorbidities and antipsychotic treatment were examined with Cox proportional hazards analysis.

**Results:**

The all-cause mortality rate was 3.9% (44 patients). Multivariate Cox regression analysis revealed that several factors were independently associated with all-cause mortality, including diabetes mellitus (DM), hypertension, heart failure, gastroesophageal reflux disease (GERD), peptic ulcer disease, ileus, underweight, fasting glucose, triglycerides, albumin, and hemoglobin. Structural equation modeling (SEM) analysis revealed that several factors had statistically significant direct effects on all-cause mortality. Heart failure, hypertension, underweight, age at onset, and ileus showed positive direct effects, while albumin and hemoglobin demonstrated negative direct effects. In addition, several factors had indirect effects on all-cause mortality. GERD indirectly affected all-cause mortality through ileus, and peptic ulcer disease had indirect effects through albumin and ileus. Ileus, underweight, DM, and hypertension also exhibited indirect effects through various pathways involving albumin, hemoglobin, and heart failure. Overall, the final model, which included these factors, explained 13% of the variability in all-cause mortality.

**Discussion:**

These results collectively suggest that the presence of DM, hypertension, heart failure, GERD, peptic ulcer disease, ileus, and underweight, along with lower levels of albumin or hemoglobin, were independently associated with all-cause mortality. The SEM analysis further revealed potential causal pathways and inter-relationships among these risk factors contributing to all-cause mortality in patients with chronic schizophrenia.

## Introduction

Schizophrenia is a multidimensional disorder that encompasses various subtypes, each with distinct neurobiological underpinnings (1–3). Recognized as a chronic and severe mental disorder, schizophrenia is a psychiatric syndrome characterized by positive, negative, and disorganized symptoms (4). While schizophrenia has an estimated heritability of 79%, it can also be influenced by various factors, including genetics, environment, exposure to viruses, infection, prenatal malnutrition, birth complications, psychosocial factors, migrant status, and urbanicity (5–7). The highest burden of schizophrenia is observed among individuals aged 25–54 years, encompassing their most productive years (8).

Schizophrenia is a global disorder (9), affecting an estimated 24 million people [~1 in 300 people (0.32%)] worldwide. Among adults, the prevalence of schizophrenia is estimated to be 1 in 222 people (0.45%) according to the Institute of Health Metrics and Evaluation (10). With a lifetime prevalence ranging from approximately 0.5% to 1%, schizophrenia is a significant global public health concern (11). According to the Global Burden of Disease 2019, the raw prevalence, incidence, and burden of schizophrenia have increased since 1990, and no reduction has been observed in age-adjusted estimates (12). Regional studies in the United States, China, India, and South Korea have suggested a steady increase in the annual incidence of schizophrenia (13–15). In Taiwan, the 1-year prevalence rate of schizophrenia is 3.34 per 1,000 people, with a 6-year (cumulative) prevalence rate of 6.42 per 1,000 people from 1996 to 2001 (16). Of all psychiatric disorders, schizophrenia is associated with one of the highest risks of mortality (17), with an all-cause mortality rate 2 to 3-fold higher than that in the general population, and a substantially shorter life expectancy (18–20). Consequently, studies on the risk factors affecting mortality in patients with chronic schizophrenia are warranted.

The modifiable risk factors associated with mortality in patients with schizophrenia include limited access to physical care, poor lifestyle behaviors, and whether or not antipsychotic medications are prescribed (21, 22). Schizophrenia has also been associated with elevated frequencies of comorbidities, with the majority of excess deaths being attributed to chronic diseases including type II diabetes mellitus (DM) (23), hypertension (24), cardiovascular diseases (25), respiratory diseases (26), stroke (27), and cancer (28), with unnatural causes such as suicide accounting for <15% (29). In addition, a previous study demonstrated a high cardiometabolic risk in patients with schizophrenia spectrum disorders. This underscores the importance of proper management, ranging from lifestyle modifications to addressing risk factors, and including the careful selection of antipsychotic drugs with a favorable cardiometabolic profile (30). However, few studies have investigated the effect of demographics, clinical characteristics, lifestyle factors, comorbid illnesses, and biochemical factors on all-cause mortality in patients with chronic schizophrenia. Moreover, Peritogiannis et al. demonstrated a complex interplay of factors that synergistically contribute to physical morbidity in patients with chronic schizophrenia, ultimately leading to increased mortality (31). Furthermore, another study reported that the association between schizophrenia and cardiometabolic risk factors is complex and influenced by an interplay of environmental factors, genetic vulnerability, and disease-related factors (32). Thus, we hypothesized that to comprehend the effects of inter-relationships and causal pathways of risk factors on all-cause mortality in patients with chronic schizophrenia, various aspects need to be considered.

To test this hypothesis, the aims of this study were: (1) to conduct a retrospective study assessing the associations among risk factors at baseline including demographics, clinical characteristics, lifestyle factors, comorbid illnesses [e.g. DM, chronic kidney disease (CKD), hypertension, gastrointestinal diseases, liver diseases, heart failure, anemia, cardiovascular diseases, cardiac arrhythmias, chronic obstruction pulmonary disease/asthma, cancer, peripheral arterial occlusion disease, obesity status], and biochemical factors with all-cause mortality in patients with chronic schizophrenia; and (2) use a structural equation model (SEM) to determine possible causal inter-relationships among the aforementioned risk factors and all-cause mortality. In addition, to emphasize the importance of regular screening for risk factors in patients with chronic schizophrenia.

## Methods and materials

### Patients

In this retrospective study, we conducted an analysis of 1,684 consecutive patients using the electronic database of Taipei Veterans General Hospital, Yuli Branch, from April 2003 to August 2022. The entire dataset was de-identified, delinked, and encrypted before being made available for analysis. The inclusion criteria were patients with: (1) chronic schizophrenia who were >18 years of age at the diagnosis, (2) schizophrenia diagnosed according to the Diagnostic and Statistical Manual of Mental Disorders IV, (3) complete clinical and follow-up data. The exclusion criteria were: (1) a diagnosis of major affective disorders (including mania, major depression, bipolar disorder and schizoaffective disorder), and (2) patients without complete clinical and follow-up data. In addition, as our psychiatric ward does not admit patients with substance and alcohol abuse or dependence, these patients were not included in this study. Moreover, patients with a poor treatment response and those with suicidal intention or behavior were also excluded from the study to mitigate the impact of these factors. Although some of the patients had a history of treatment resistance or suicidal behavior, those included in this study were in a stable and chronic condition. In our hospital, we use the Clinical Global Impression Severity (CGI-S) score (33) and Mini-Mental State Examination (MMSE) test to assess the psychological and intellectual clinical status of patients. The CGI-S is rated on a 7-point scale as follows: 1 = normal, not at all ill; 2 = borderline mentally ill; 3 = mildly ill; 4 = moderately ill; 5 = markedly ill; 6 = severely ill; 7 = among the most extremely ill patients. In general, none of the patients at our institution have a low CGI-S score, because all of our patients are chronic and have been transferred from other psychiatric wards in Taiwan. Those with higher CGI-S scores were initially admitted to the acute ward and subsequently excluded from our study. Consequently, the CGI-S scores of the patients included in this study at the time of inclusion ranged between 3 and 4, which is equivalent to positive and negative syndrome scale scores of 55 to 78. In addition, the median MMSE score (interquartile range) of the patients was 23.0 (17.0-27.0) (Table 1).

**Table 1:** Baseline clinical characteristics of the study participants by survival status.

**Variable**	**Total**	**Death**	**Survival**	**χ^2^**	***df***	***p*-value**	**Effect size**
No	1,126	44	1,082				
Age	56.7 ± 11.7	66.6 ± 12.6	56.3 ± 11.5	-	-	< 0.0001	-
Female gender	540 (48.0)	29 (65.9)	511 (47.2)	5.912	1	0.015	0.07
Age at onset	24.0 ± 10.6	31.5 ± 17.6	23.7 ± 10.1	-	-	< 0.0001	-
MMSE score	23.0 (17.0-27.0)	19.0 (15.5-27.5)	23.0 (17.0-27.0)	-	-	0.318	-
Systolic blood pressure	123 ± 15	120 ± 18	123 ± 15	-	-	0.256	-
Diastolic blood pressure	75 ± 11	70 ± 12	76 ± 11	-	-	0.001	-
Body mass index	23.7 ± 4.7	19.2 ± 5.5	23.9 ± 4.6	-	-	< 0.0001	-
Smoking	347 (30.8)	8 (18.2)	339 (31.3)	3.442	1	0.064	−0.06
Drinking	253 (22.5)	6 (13.6)	247 (22.8)	2.059	1	0.151	−0.04
Diabetes mellitus	196 (17.4)	13 (29.6)	183 (16.9)	4.678	1	0.031	0.07
Hypertension	410 (36.4)	26 (59.1)	384 (35.5)	10.139	1	0.002	0.10
Hyperlipidemia	209 (18.6)	10 (22.7)	199 (18.4)	0.521	1	0.470	0.02
Anemia	231 (20.5)	17 (38.6)	214 (19.8)	9.197	1	0.002	0.09
Chronic kidney disease	51 (4.5)	3 (6.8)	48 (4.4)	0.552	1	0.457	0.02
Cardiovascular disease	57 (5.1)	5 (11.4)	52 (4.8)	3.775	1	0.052	0.06
Heart failure	23 (2.0)	5 (11.4)	18 (1.7)	19.858	1	< 0.0001	0.13
Cardiac arrhythmias	27 (2.4)	2 (4.6)	25 (2.3)	0.900	1	0.343	0.03
PAOD	7 (0.6)	1 (2.3)	6 (0.6)	2.017	1	0.156	0.04
GERD	146 (13.0)	13 (29.6)	133 (12.3)	11.129	1	0.001	0.10
Peptic ulcer disease	65 (5.8)	6 (13.6)	59 (5.5)	5.195	1	0.023	0.07
Ileus	82 (7.3)	9 (20.5)	73 (6.8)	11.745	1	0.001	0.10
Cancer	72 (6.4)	6 (13.6)	66 (6.1)	4.003	1	0.045	0.06
HBV/HCV	154 (13.7)	11 (25.0)	143 (13.2)	4.959	1	0.026	0.07
COPD/asthma	58 (5.2)	2 (4.6)	56 (5.2)	0.035	1	0.852	−0.01
**Obesity status**							
Underweight	121 (10.8)	20 (45.5)	101 (9.3)	58.889	1	< 0.0001	0.25
Normal weight	428 (38.0)	16 (36.4)	412 (38.1)	0.013	1	0.911	−0.003
Overweight	250 (22.2)	3 (6.8)	247 (22.8)	5.970	1	0.015	−0.08
Mild obesity	164 (14.6)	1 (2.3)	163 (15.1)	5.393	1	0.020	−0.07
Moderate and severe obesity	163 (14.5)	3 (6.8)	160 (14.8)	2.028	1	0.154	−0.04
**Antipsychotics**							
Typical antipsychotics	382 (33.9)	16 (36.4)	366 (33.8)	0.255	1	0.614	0.02
Atypical antipsychotics	744 (66.1)	28 (63.6)	716 (66.2)				

Data are presented as means ± SD, number (percentage), or median (interquartile range). MMSE, Mini-Mental State Examination; PAOD, peripheral arterial occlusion disease; GERD, gastroesophageal reflux disease; HBV, hepatitis B virus; HCV, hepatitis C virus; COPD, chronic obstruction pulmonary disease.

### Data collection

Clinical information collected for subsequent analysis included: (1) sociodemographic factors: age, sex, and age at onset; (2) lifestyle factors: former/current smokers compared to non-smokers [ceased smoking ≥1 year], and former/current alcohol drinkers compared to non-drinkers [ceased drinking ≥1 year]; (3) anthropometric variables: weight, height, body mass index (BMI); and blood pressure (after a 5-min rest); (4) comorbidities: DM, hypertension, hyperlipidemia, anemia, CKD, cardiovascular diseases, heart failure, cardiac arrhythmias, peripheral arterial occlusion disease, gastroesophageal reflux disease (GERD), peptic ulcer disease, Ileus, cancer, hepatitis B virus (HBV)/hepatitis C virus (HCV) infections, chronic obstruction pulmonary disease/asthma; (5) obesity status according to the Ministry of Health and Welfare, Taiwan, definitions (all values given as BMI in kg/m^2^) (34): severe obesity (≥35), moderate obesity (30≤-<35), mild obesity (27≤-<30), overweight (24≤-<27), normal weight (18.5≤-<24), underweight (<18.5); (6) antipsychotic treatment: (typical antipsychotics and atypical antipsychotics); and (7) biochemical data: HbA1C, fasting glucose, albumin, total protein, uric acid, lipid profile, liver and renal function parameters, total and differential leukocyte counts, and hematological parameters.

Detailed data were retrieved from Taipei Veterans General Hospital, Yuli Branch Psychiatric Database. Two research assistants (P.-L.L. and P.-Y.T.) collected the data, all of which were checked by a study author (F.-M.C.).

### Ethical considerations

This study was conducted according to the principles of the Declaration of Helsinki and was approved by the Human Research Ethics Committee of Kaohsiung E-Da Hospital (EMRP61110N and EMRP66111N). To ensure the protection of any potentially identifiable personal data of the subjects, the entire dataset has been de-identified and encrypted before being made available for analysis. This process aligns with national legislation and institutional requirements. As such, written informed consent from the participants was not required.

### Statistical analysis

Categorical variables are given as frequency (percentage), while continuous variables are given as mean (±standard deviation). Comparisons in baseline variables between survivors and non-survivors were performed with the Student's *t* test or χ^2^ test. We defined the outcome as the duration from diagnosis to death. Univariate and multivariate Cox proportional hazard analyses were performed to evaluate relationships between baseline biochemical and clinical risk factors with all-cause mortality, and the results are presented as hazard ratio (HR) with 95% confidence interval (CI). JMP (version 7.0, SAS Institute) was used for all other statistical analyses. Two-sided *p* < 0.05 were considered significant. In addition, we also used IBM SPSS AMOS version 24 (Amos Development Corporation, Meadville, PA, USA) to fit the path model and SEM. We used root mean square error of approximation <0.08, standardized root mean square residual <0.06, and comparative fit index (CFI) >0.90 to assess the fit of the data to the models (35). Furthermore, model fit was estimated using the maximum likelihood method. The findings are presented as standardized path coefficients along with the corresponding statistical significance.

## Results

Of the 1,684 patients with chronic schizophrenia initially screened from April 2003 to August 2022, 558 were excluded from the study (90 with higher CGI-S scores and 468 without complete clinical and follow-up data). The final study population included 1,126 consecutive patients (586 men and 540 women; mean age, 56.7 ± 11.7 years), and they were followed until October 31, 2022 (median follow-up, 26.3 months; range, 2–230 months). At the end of the study, 44 patients (3.9%) had died of all causes, of whom four were related to choking, three to coronavirus disease 2019 (COVID-19) infection, one to hepatocellular carcinoma, 21 to sepsis due to pneumonia and urinary tract infections, and 15 to sudden cardiac death.

### Baseline characteristics

At baseline, the median age of the patients was 57 (range, 23–94) years, 196 (17.4%) patients had DM, 209 (18.6%) had hyperlipidemia, and 410 (36.4%) had hypertension (Table 1). The non-survivors were older, had an older age at onset, and lower diastolic blood pressure and BMI values than the survivors. In addition, more of the non-survivors were female, and they had higher rates of DM, hypertension, anemia, heart failure, GERD, peptic ulcer disease, ileus, cancer, HBV and HCV infections, and underweight, and lower rates of overweight and mild obesity than the survivors (Table 1).

### Baseline biochemical characteristics

The non-survivors had higher baseline alkaline phosphatase and eosinophil count, and lower fasting glucose, total cholesterol, LDL-cholesterol, albumin, lymphocyte count, red blood cell count, hemoglobin, and hematocrit than the survivors (Table 2).

**Table 2:** Baseline biochemical data of the study participants by survival status.

**Variable**	**Total**	**Death**	**Survival**	***p*-value**
No	1,126	44	1,082	
HbA1C (%)	5.8 ± 0.7	5.8 ± 0.5	5.8 ± 0.8	0.782
Fasting glucose (mg/dL)	94.7 ± 28.0	82.8 ± 17.7	95.2 ± 28.3	0.005
Total cholesterol (mg/dL)	153.3 ± 31.8	140.2 ± 34.6	153.8 ± 31.6	0.007
Triglycerides (mg/dL)	97.0 ± 53.9	84.7 ± 39.0	97.5 ± 54.4	0.135
HDL-cholesterol (mg/dL)	52.0 ± 14.5	53.8 ± 16.5	51.9 ± 14.4	0.431
LDL-cholesterol (mg/dL)	88.9 ± 27.5	78.2 ± 22.0	89.3 ± 27.6	0.011
Albumin (g/dL)	4.0 ± 0.4	3.5 ± 0.7	4.0 ± 0.4	< 0.0001
Total protein (g/dL)	7.1 ± 0.7	6.7 ± 0.9	7.1 ± 0.7	0.079
Uric acid (mg/dL)	4.8 ± 1.7	4.3 ± 1.8	4.8 ± 1.7	0.079
Aspartate aminotransferase (U/L)	21.0 ± 13.1	21.9 ± 9.9	20.9 ± 13.3	0.643
Alanine aminotransferase (U/L)	13.0 (9.0–19.0)	12.5 (8.0–20.0)	13.0 (9.0–19.0)	0.945
Alkaline phosphatase (U/L)	86.5 ± 34.5	107.2 ± 37.8	85.2 ± 34.0	0.021
Creatinine (mg/dL)	0.84 ± 0.42	0.79 ± 0.36	0.84 ± 0.42	0.478
eGFR (ml/min/1.73 m^2^)	119.1 ± 41.1	126.5 ± 54.7	118.8 ± 40.4	0.230
White blood cell (× 10^9^/L)	6.490 ± 2.421	6.316 ± 1.978	6.497 ± 2.438	0.627
Neutrophil count (× 10^9^/L)	4,056 ± 2,273	4,138 ± 1,771	4,052 ± 2,291	0.811
Monocyte count (× 10^9^/L)	407 ± 254	406 ± 161	407 ± 257	0.991
Lymphocyte count (× 10^9^/L)	1,824 ± 712	1,468 ± 545	1,838 ± 715	0.001
Eosinophil count (× 10^9^/L)	136 (73–230)	180 (100–316)	132 (72–226)	0.008
Basophil count (× 10^9^/L)	33.9 ± 19.2	29.8 ± 17.4	34.1 ± 19.3	0.154
Red blood cell (10^∧6^/μL)	4.2 ± 0.6	3.8 ± 0.6	4.2 ± 0.6	0.0001
Hemoglobin (g/dL)	12.5 ± 1.6	11.2 ± 1.6	12.5 ± 1.6	< 0.0001
Hematocrit (%)	36.4 ± 4.5	32.4 ± 6.5	36.6 ± 4.3	< 0.0001
Mean corpuscular volume (fL)	87.9 ± 6.7	86.8 ± 7.6	87.9 ± 6.7	0.284
Platelet (10^∧^3/μL)	231.8 ± 73.7	235.8 ± 81.1	231.7 ± 73.4	0.713

Data are means ± SD or median (interquartile range). HDL-C, high-density lipoprotein cholesterol; LDL-C, low-density lipoprotein cholesterol; eGFR, estimated glomerular filtration rate.

### Associations of the baseline clinical variables with all-cause mortality

Univariate Cox regression analysis showed that age, age at onset, diastolic blood pressure, BMI, DM, hypertension, anemia, heart failure, GERD, peptic ulcer disease, ileus, HBV/HCV infections, and underweight were associated with all-cause mortality. Multivariate Cox regression analysis showed that DM [HR 4.02 (1.83–8.52), *p* = 0.001], hypertension [HR 3.45 (1.66–7.35), *p* = 0.001], heart failure [HR 4.31 (1.13–12.90), *p* = 0.035], GERD [HR 2.48 (1.13–5.19), *p* = 0.025], peptic ulcer disease [HR 3.18 (1.15–7.50), *p* = 0.028], ileus [HR 2.80 (1.19–6.01), *p* = 0.020], and underweight [HR 3.91 (1.10–18.46), *p* = 0.034] were independently associated with all-cause mortality (Table 3).

**Table 3:** Cox proportional hazard model of baseline clinical risk factors for the development of all-cause mortality in the whole cohort.

**Baseline data**	**Univariate analysis HR (95% CI)**	***p*-value**	Multivariate analysis HR (95% CI)^*^	***p*-value**
Age	1.08 (1.05–1.11)	< 0.0001	-	-
Female gender	1.69 (0.91–3.25)	0.098	-	-
Age at onset	1.04 (1.02–1.06)	< 0.0001	-	-
Systolic blood pressure	0.98 (0.96–1.00)	0.097	-	-
Diastolic blood pressure	0.96 (0.94–0.99)	0.002	-	-
Body mass index	0.75 (0.70–0.82)	< 0.0001	-	-
Smoking	0.56 (0.24–1.15)	0.119	-	-
Drinking	0.58 (0.22–1.28)	0.190	-	-
Diabetes mellitus	2.17 (1.09–4.07)	0.028	4.02 (1.83–8.52)	0.001
Hypertension	3.28 (1.79–6.17)	0.0001	3.45 (1.66–7.35)	0.001
Hyperlipidemia	1.41 (0.66–2.75)	0.360	1.89 (0.80–4.09)	0.139
Anemia	2.92 (1.55–5.35)	0.001	1.91 (0.90–3.98)	0.092
Chronic kidney disease	2.06 (0.50–5.72)	0.276	1.50 (0.24–5.08)	0.604
Cardiovascular disease	2.40 (0.72–5.96)	0.139	1.25 (0.35–3.44)	0.709
Heart failure	9.81 (3.35–9.07)	0.0003	4.31 (1.13–12.90)	0.035
Cardiac arrhythmias	1.96 (0.32–6.37)	0.401	1.33 (0.21–4.73)	0.715
PAOD	4.05 (0.23–8.68)	0.259	1.85 (0.09–12.24)	0.623
GERD	4.09 (2.05–7.77)	0.0002	2.48 (1.13–5.19)	0.025
Peptic ulcer disease	3.35 (1.26–7.40)	0.018	3.18 (1.15–7.50)	0.028
Ileus	4.62 (2.06–9.35)	0.001	2.80 (1.19–6.01)	0.020
Cancer	2.34 (0.89–5.16)	0.081	1.67 (0.58–4.13)	0.321
HBV/HCV	2.32 (1.11–4.48)	0.026	1.58 (0.68–3.37)	0.271
COPD/asthma	1.05 (0.17–3.43)	0.945	1.26 (0.19–4.56)	0.773
**Obesity status**				
Underweight	11.02 (3.77–16.81)	< 0.0001	3.91 (1.10–18.46)	0.034^†^
Normal weight	2.00 (0.66–8.63)	0.239	0.74 (0.22–3.43)	0.671^†^
Overweight	0.64 (0.12–3.45)	0.585	0.31 (0.05–1.79)	0.181^†^
Mild obesity	0.34 (0.02–2.64)	0.312	0.25 (0.01–1.97)	0.194^†^
Moderate and severe obesity	Ref		Ref	

^*^Adjusted for age, sex, age at onset, systolic blood pressure, diastolic blood pressure, body mass index, smoking, and drinking. ^†^Adjusted for age, sex, age at onset, systolic blood pressure, diastolic blood pressure, smoking, and drinking. PAOD, peripheral arterial occlusion disease; GERD, gastroesophageal reflux disease; HBV, hepatitis B virus; HCV, hepatitis C virus; COPD, chronic obstruction pulmonary disease.

### Associations of the baseline biochemical variables with all-cause mortality

Univariate Cox regression analysis showed that fasting glucose, total cholesterol, LDL-cholesterol, albumin, lymphocyte count, red blood cell count, hemoglobin, and hematocrit were associated with all-cause mortality. Multivariate Cox regression analysis showed that fasting glucose [HR 0.96 (0.93–0.99), *p* = 0.044], triglycerides [HR 0.98 (0.96–0.99), *p* = 0.048], albumin [HR 0.10 (0.03–0.28), *p* < 0.0001], and hemoglobin [HR 0.33 (0.12–0.92), *p* = 0.035] were independently associated with all-cause mortality (Table 4).

**Table 4:** Cox proportional hazard model of baseline biochemical risk factors for the development of all-cause mortality in the whole cohort.

**Baseline data**	**Univariate analysis HR (95% CI)**	***p*-value**	**Multivariate analysis HR (95% CI)**	***p*-value**
HbA1C	0.88 (0.49–1.38)	0.613		
Fasting glucose	0.96 (0.94–0.98)	< 0.0001	0.96 (0.93–0.99)	0.044
Total cholesterol	0.98 (0.97–0.99)	0.002		
Triglycerides	0.99 (0.99–1.00)	0.072	0.98 (0.96–0.99)	0.048
HDL-cholesterol	1.00 (0.98–1.02)	0.795		
LDL-cholesterol	0.98 (0.97–0.99)	0.003		
Albumin	0.13 (0.06–0.30)	< 0.0001	0.10 (0.03–0.28)	< 0.0001
Total protein	0.37 (0.13–1.00)	0.051		
Uric acid	0.88 (0.72–1.07)	0.198		
Aspartate aminotransferase	1.00 (0.98–1.01)	0.627		
Alanine aminotransferase	1.00 (0.98–1.01)	0.890		
Alkaline phosphatase	1.01 (0.99–1.02)	0.067		
Creatinine	0.83 (0.22–1.70)	0.709		
eGFR	1.00 (0.99–1.01)	0.307		
White blood cell	0.99 (0.99–1.00)	0.796		
Neutrophil count	1.00 (0.99–1.00)	0.699		
Monocyte count	1.00 (0.99–1.00)	0.970		
Lymphocyte count	0.99 (0.99–1.00)	0.001		
Eosinophil count	1.00 (0.99–1.00)	0.077		
Basophil count	0.99 (0.97–1.01)	0.154		
Red blood cell	0.35 (0.21–0.59)	0.0001		
Hemoglobin	0.58 (0.48–0.70)	< 0.0001	0.33 (0.12–0.92)	0.035
Hematocrit	0.87 (0.84–0.91)	< 0.0001		
Mean corpuscular volume	0.97 (0.93–1.01)	0.149		
Platelet	1.00 (0.99–1.00)	0.894		

HDL-C, high-density lipoprotein cholesterol; LDL-C, low-density lipoprotein cholesterol; eGFR, estimated glomerular filtration rate. Multivariate stepwise Cox regression analysis including all the variables that demonstrated a p-value < 0.1 in Univariate analysis listed in the table.

### SEM analysis

**Figure 1:**
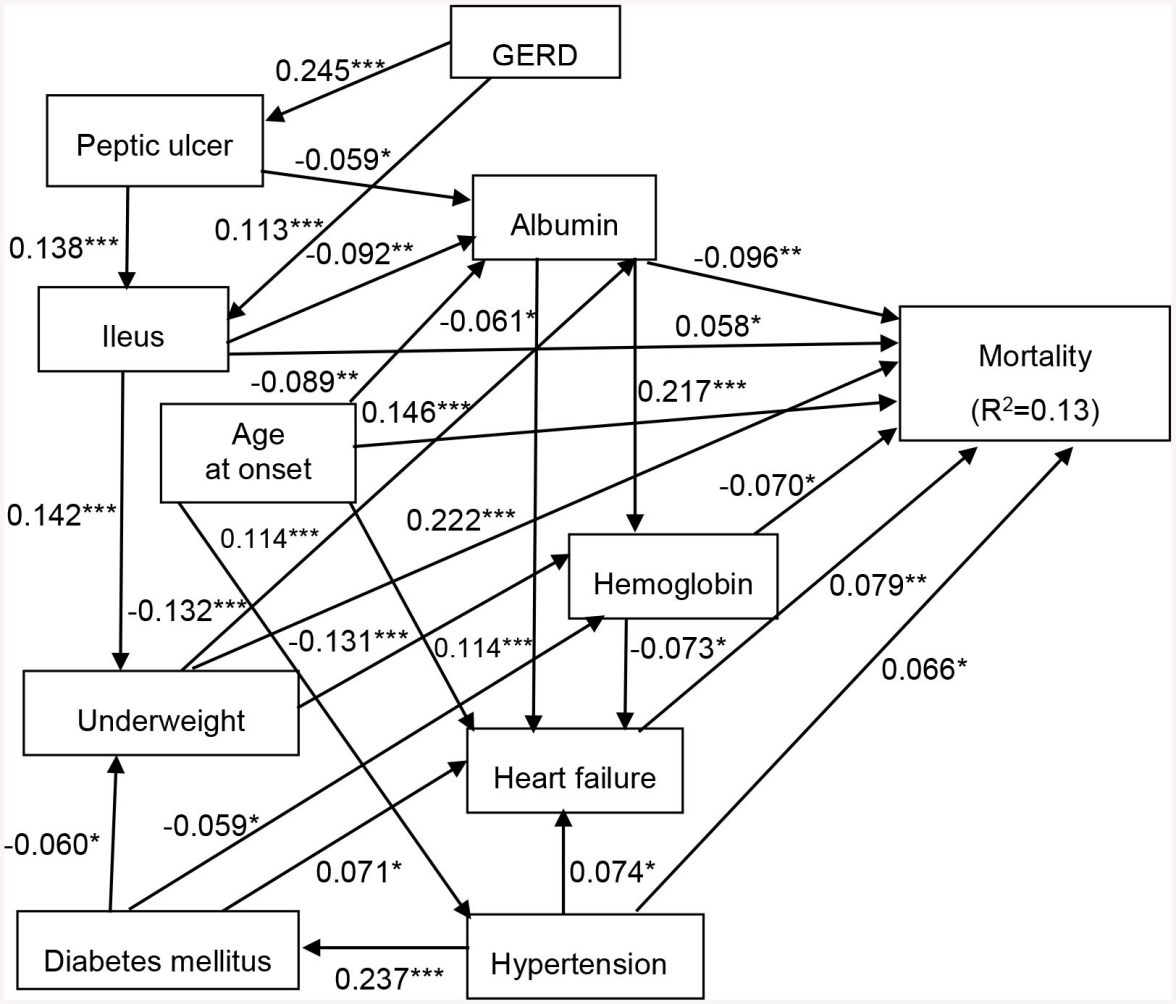
Structural equation model for all-cause mortality in patients with chronic schizophrenia. Comparative fit index (CFI), 0.958; goodness of fit index (GFI), 0.992; root mean square error of approximation (RMSEA), 0.027; standardized root mean square residual (SRMR), 0.030. ^*^p < 0.05, ^**^p < 0.01, and ^***^p < 0.001. Path loadings are standardized coefficients. GERD, gastroesophageal reflux disease.

As with the Cox proportional hazard model described above (Tables 3, 4), we designed an SEM model to assess the effects of DM, hypertension, heart failure, GERD, peptic ulcer disease, ileus, underweight, albumin, and hemoglobin on all-cause mortality. The results showed that the model fit the data well, with a CFI of 0.958, a root mean square error of approximation of 0.027, and a standardized root mean square residual of 0.030 (Figure 1). Heart failure (β = 0.079), hypertension (β = 0.066), underweight (β = 0.222), age at onset (β = 0.146), and ileus (β = 0.058) had statistically significant positive direct effects on all-cause mortality. In addition, albumin (β = −0.096) and hemoglobin (β = −0.070) had statistically significant negative direct effects on all-cause mortality. Moreover, GERD indirectly affected all-cause mortality through ileus (β = 0.113). Peptic ulcer disease indirectly affected all-cause mortality through albumin (β = −0.059) and ileus (β = 0.138). Ileus indirectly affected all-cause mortality through albumin (β = −0.092) and underweight (β = 0.142). Underweight indirectly affected all-cause mortality through albumin (β = −0.132) and hemoglobin (β = −0.131). DM indirectly affected all-cause mortality through heart failure (β = 0.071) and hemoglobin (β = −0.059). Age at onset indirectly affected all-cause mortality through albumin (β = −0.089), heart failure (β = 0.114), and hypertension (β = 0.114). Albumin indirectly affected all-cause mortality through hemoglobin (β = 0.217) and heart failure (β = −0.061). Hemoglobin indirectly affected all-cause mortality through heart failure (β = −0.073). Hypertension indirectly affected all-cause mortality through heart failure (β = 0.074). The model explained 13% of the variability in all-cause mortality (Figure 1).

## Discussion

In this study, we investigated associations among baseline risk factors with all-cause mortality in patients with chronic schizophrenia. There are two key findings in this study. First, multivariate Cox regression analysis showed that DM, hypertension, heart failure, GERD, peptic ulcer disease, ileus, underweight, fasting glucose, triglycerides, albumin, and hemoglobin all contributed to the risk of all-cause mortality in the enrolled patients with chronic schizophrenia. Second, the causal relationships of DM, hypertension, heart failure, GERD, peptic ulcer disease, ileus, underweight, albumin, and hemoglobin on all-cause mortality were confirmed by SEM analysis.

A previous study reported a 2.9-fold higher risk of all-cause mortality in patients with schizophrenia compared to the general population, and a 1.6-fold higher risk compared to controls matched for physical diseases (36). Although several risk factors including cardiovascular diseases (37), stroke (37, 38), smoking (39), physical fitness and inactivity (40), obesity (41), dyslipidemia (42), any cancer (43), and suicide (44) have been associated with all-cause mortality in patients with schizophrenia, the exact pathophysiological mechanisms have yet to be clarified.

We also found associations between all-cause mortality with DM, hypertension, heart failure, GERD, peptic ulcer disease, ileus, underweight, fasting glucose, triglycerides, albumin, and hemoglobin in our study cohort (Tables 3, 4). Previous studies have reported a 2 to 3-fold higher prevalence of type 2 DM in patients with schizophrenia compared to the general population, with a rate ranging from 6% to 21% (45, 46). Patients with schizophrenia have a high mortality rate, and metabolic abnormalities including type 2 DM are important causes. Possible etiologies of the development of diabetes in schizophrenic patients include: (a) inherited susceptibility to both schizophrenia and diabetes (47); (b) antipsychotic medications that affect dopaminergic, serotonergic, and histaminergic receptors, and hypothalamic regulation (48), and influence leptin resistance and pancreatic muscarinic receptors (48); (c) neuroendocrine pathways, elevated cortisol and hypothalamic axis dysregulation (49), and nutritional deficiency (50, 51); (d) many environmental factors, such as limited availability of quality food and poor diet (52), and insufficient physical activity due to social isolation and symptoms (52, 53), which are related to both diabetes and schizophrenia. However, we found that lower fasting glucose and triglycerides were associated with higher all-cause mortality in our study cohort. Consistent with our results, a previous study reported a significantly higher prevalence of hypotriglyceridemia in underweight schizophrenic patients (54). The poor diet in patients with schizophrenia could partially explain the higher prevalence of metabolic abnormalities (55).

A previous study reported a possible link between severe mental illnesses including schizophrenia with an increased risk of alterations in left ventricular function and structure due to the early onset of cardiovascular disease and other factors including smoking, obesity, hypertension, myocardial infarction and DM (56). These factors could lead to a greater decrease in left ventricular ejection fraction and more severe heart failure compared to the general population (57, 58). In addition, in a study of approximately 22,000 schizophrenic patients, Kilbourne et al. reported that hypertension was a major risk factor for cardiac death (59). These findings support the association between hypertension and heart failure with all-cause mortality in the present study (Table 3). Similarly, we also found that peptic ulcer disease, GERD, and ileus were related to all-cause mortality. This is in agreement with other studies which have reported an association between schizophrenia with peptic ulcer disease, GERD, and ileus (60–62). Liao et al. suggested that schizophrenic patients are at a slightly elevated risk of peptic ulcer disease compared to the general population (60). This may be due to higher rates of *Helicobacter pylori* infection, smoking, alcohol consumption, taking anxiolytics and hypnotics, anti-depressants, or non-steroidal anti-inflammatory drugs among these patients. Furthermore, Kasap et al. suggested that schizophrenic patients who smoke and drink alcohol may have a higher rate of reflux symptoms (61). Moreover, Nielsen et al. suggested that female sex, older age, treatment with high-potency first-generation antipsychotic drugs, clozapine, anticholinergics, tricyclic antidepressants, and opioids may be associated with a higher risk of ileus in patients with schizophrenia (62). In the present study, we also found that underweight status was related to all-cause mortality. This finding was also reported in a previous study, in which all-cause mortality was associated with underweight status compared with normal weight status (HR: 1.33, 95% CI: 1.01–1.76), potentially due to frailty in older age groups (41).

Multivariate Cox regression analysis of the biochemical risk factors in this study showed associations between albumin and hemoglobin with all-cause mortality (Table 4). Huang reported a significantly lower serum albumin level in Taiwanese schizophrenic patients during the acute phase compared to controls (63). In addition, serum albumin level has been proposed to be a prognostic indicator of mortality in older hospitalized patients (64), survival in women infected with immunodeficiency virus (65), and disease in patients with inflammation or injury (66). Regarding the association between hemoglobin and all-cause mortality, a previous study reported a higher prevalence of anemia among chronic psychiatric patients compared to the general population (67). This could be due to reasons including poor physical condition and lifestyle habits, drugs taken, and nutritional disorders. This may suggest that serum albumin and hemoglobin concentrations could also be used as markers of the clinical course in patients with schizophrenia.

To the best of our knowledge, this study is the first to investigate the causal relationships of DM, hypertension, heart failure, GERD, peptic ulcer disease, ileus, underweight, albumin, and hemoglobin with all-cause mortality in patients with schizophrenia. However, the exact mechanisms underlying the associations among these risk factors with all-cause mortality remain unclear. SEM analysis showed significant positive direct effects from heart failure, underweight, age at onset, ileus and hypertension on all-cause mortality. Furthermore, albumin and hemoglobin had significant negative direct effects on all-cause mortality. Previous studies have demonstrated associations between DM (36), hypertension (59), heart failure (68), and gastrointestinal diseases (e.g., GERD, peptic ulcer disease, and ileus) (36), underweight (41), age at onset (36), albumin (64), and hemoglobin (67) with all-cause mortality in patients with schizophrenia. We also found that GERD indirectly affected all-cause mortality through ileus, peptic ulcer disease indirectly affected all-cause mortality through low albumin and ileus, and that ileus indirectly affected all-cause mortality through low albumin and underweight. In addition, we found that underweight indirectly affected all-cause mortality through low albumin and low hemoglobin, DM and albumin indirectly affected all-cause mortality through heart failure and low hemoglobin, and that hemoglobin and hypertension indirectly affected all-cause mortality through heart failure. The most frequent clinical signs of ileus are a decrease in or no intestinal sounds and gastric reflux (69). Nielsen et al. showed that clozapine or anticholinergic treatment was associated with a higher risk of fatal ileus in patients with schizophrenia (62). Furthermore, emerging evidence has suggested relationships between a lower serum albumin level with peptic ulcer disease and bowel disease in patients with schizophrenia (63, 70). Notably, a previous study (63) reported that Taiwanese inpatients with schizophrenia had lower serum albumin levels, suggesting that patients in the acute phase of disease have similar systemic responses, as also shown in other studies (71, 72). Moreover, the combination of hypoalbuminemia and low BMI has been proposed to potentially be a useful marker of high mortality in older people (73). Kamruzzaman found that underweight women were more likely to be anemic (74). In addition, anemia has been shown to have a cumulative additive effect on left ventricular function and global strain in patients with type 2 DM (75). Furthermore, anemia is a common comorbidity in patients with heart failure, and is associated with poor outcomes (76). Chronic hypertension and cardiac structural and functional changes can predispose to the development of heart failure (77). Therefore, it is reasonable to suggest that GERD, peptic ulcer disease, ileus, underweight, DM, albumin, hemoglobin, and hypertension may be involved in common pathways contributing to all-cause mortality in patients with chronic schizophrenia.

### Limitations

The limitations of this study include the relatively few cases of all-cause mortality. In addition, the length of follow-up (median, 26.3 months) may be insufficient to reveal cases of late all-cause mortality. Moreover, the single-center nature of this retrospective study may limit the application of our results to other Taiwanese patients with schizophrenia. Further larger-scale studies with patients of different ethnicity and longer follow-up periods are needed to verify our findings. In addition, there was a long duration between the age at onset and the start of follow-up. This extended duration raises the possibility that factors influencing mortality patterns along the treatment trajectory in individuals with schizophrenia may not align with the highest risk period for suicide in this population, and this may have influenced our findings. Finally, only patients with stable and chronic schizophrenia were included into this study. This limitation may affect the generalizability of our results to other subtypes of schizophrenia or to patients with acute conditions and high CGI-S scores.

### Conclusions

In summary, we found that DM, hypertension, heart failure, GERD, peptic ulcer disease, ileus, underweight, lower circulating albumin and hemoglobin levels were associated with all-cause mortality in patients with chronic schizophrenia. In addition, SEM delineated inter-relationships of the risk factors and potential pathways that may contribute to all-cause mortality in patients with chronic schizophrenia. These findings provide valuable insights for improving clinical practice, and the identified risk factors could serve as important indicators for clinicians to closely monitor and manage individuals with chronic schizophrenia. Regular screening for DM, hypertension, and heart failure, along with vigilant management of gastrointestinal issues such as GERD and peptic ulcer disease may help to prevent adverse outcomes. In addition, efforts should be directed toward optimizing nutritional status, including addressing underweight and ensuring adequate levels of albumin and hemoglobin. Moreover, in terms of integrative care, a multidisciplinary approach could be beneficial, and collaborative efforts involving psychiatrists, primary care physicians, nutritionists, and other healthcare professionals would enhance the comprehensive care of individuals with schizophrenia. Integrated care models that focus on both mental and physical health, incorporating regular health assessments, lifestyle interventions, and patient education, may contribute to better outcomes. Mental health and medical providers should work together to develop personalized care plans that address the specific needs of individuals with schizophrenia, considering the identified risk factors for mortality. Furthermore, promoting patient engagement and self-management could play an important role in achieving holistic wellbeing in this population. Our findings suggest that a proactive and integrated approach to healthcare delivery, considering the complex interplay of risk factors, is essential for improving the overall health and longevity of individuals with chronic schizophrenia.

## Data availability statement

The original contributions presented in the study are included in the article/supplementary material, further inquiries can be directed to the corresponding authors.

## Ethics statement

The studies involving humans were approved by The Human Research Ethics Committee of Kaohsiung E-Da Hospital (EMRP61110N and EMRP66111N). The studies were conducted in accordance with the local legislation and institutional requirements. Written informed consent from the participants was not required to participate in this study in accordance with the national legislation and the institutional requirements.

## Author contributions

T-HY: Conceptualization, Data curation, Funding acquisition, Investigation, Methodology, Resources, Supervision, Validation, Visualization, Writing—original draft, Writing—review & editing. T-LL: Data curation, Investigation, Resources, Writing—original draft, Writing—review & editing. C-FH: Data curation, Investigation, Resources, Writing—original draft, Writing—review & editing. C-CW: Data curation, Investigation, Resources, Writing—original draft, Writing—review & editing. C-PW: Data curation, Investigation, Resources, Writing—original draft, Writing—review & editing. Y-CL: Data curation, Investigation, Resources, Writing—original draft, Writing—review & editing. C-TW: Writing—original draft, Writing—review & editing. F-MC: Formal analysis, Project administration, Writing—original draft, Writing—review & editing. Y-JL: Methodology, Visualization, Writing—original draft, Writing—review & editing. I-TT: Writing—review & editing, Conceptualization, Methodology, Validation, Visualization, Writing—original draft. W-HT: Conceptualization, Data curation, Funding acquisition, Investigation, Methodology, Resources, Supervision, Validation, Visualization, Writing—original draft, Writing—review & editing.

## References

[1] Ventriglio A Bellomo A Ricci F Magnifico G Rinaldi A Borraccino L. New pharmacological targets for the treatment of schizophrenia: a literature review. Curr Top Med Chem. (2021) 21:1500–16. 10.2174/1568026621666210701103147 34218785

[2] Ebisch SJ Mantini D Northoff G Salone A De Berardis D Ferri F. Altered brain long-range functional interactions underlying the link between aberrant self-experience and self-other relationship in first-episode schizophrenia. Schizophr Bull. (2014) 40:1072–82. 10.1093/schbul/sbt153 24191160 PMC4133668

[3] De Berardis D De Filippis S Masi G Vicari S Zuddas A. A Neurodevelopment approach for a transitional model of early onset schizophrenia. Brain Sci. (2021) 11:275. 10.3390/brainsci11020275 33672396 PMC7926620

[4] Jauhar S Johnstone M McKenna PJ. Schizophrenia. Lancet. (2022) 399:473–86. 10.1016/S0140-6736(21)01730-X 35093231

[5] National Institute of Mental Health. Schizophrenia. Available online at: https://www.nimh.nih.gov/health/topics/schizophrenia/index.shtml (accessed February 1, 2016).

[6] Sutterland AL Fond G Kuin A Koeter MW Lutter R van Gool T. Beyond the association. Toxoplasma gondii in schizophrenia, bipolar disorder, and addiction: systematic review and meta-analysis. Acta Psychiatr Scand. (2015) 132:161–79. 10.1111/acps.12423 25877655

[7] Henssler J Brandt L Müller M Liu S Montag C Sterzer P. Migration and schizophrenia: metaanalysis and explanatory framework. Eur Arch Psychiatry Clin Neurosci. (2020) 270:325–35. 10.1007/s00406-019-01028-7 31161262

[8] Charlson FJ Ferrari AJ Santomauro DF Diminic S Stockings E Scott JG. Global epidemiology and burden of schizophrenia: findings from the global burden of disease study 2016. Schizophr Bull. (2018) 44:1195–203. 10.1093/schbul/sby058 29762765 PMC6192504

[9] Picchioni MM Murray RM. Schizophrenia. BMJ. (2007) 335:91–5. 10.1136/bmj.39227.616447.BE 17626963 PMC1914490

[10] Institute of health Metrics and Evaluation (IHME). Global Health Data Exchange (GHDx). http://ghdx.healthdata.org/gbd-results-tool?params=gbd-api-2019-permalink/27a7644e8ad28e739382d31e77589dd7 (accessed September 25, 2021).

[11] Simeone JC Ward AJ Rotella P Collins J Windisch R. An evaluation of variation in published estimates of schizophrenia prevalence from 1990-2013: a systematic literature review. BMC Psychiatry. (2015) 15:193. 10.1186/s12888-015-0578-7 26263900 PMC4533792

[12] Solmi M Seitidis G Mavridis D Correll CU Dragioti E Guimond S. Incidence, prevalence, and global burden of schizophrenia-data, with critical appraisal, from the Global Burden of Disease (GBD) 2019. Mol Psychiatry. (2023). 10.1038/s41380-023-02138-4. [Epub ahead of print]. 37500825

[13] Martin A Bessonova L Hughes R Doane MJ O'Sullivan AK Snook K. Systematic review of real-world treatment patterns of oral antipsychotics and associated economic burden in patients with schizophrenia in the United States. Adv Ther. (2022) 39:3933–56. 10.1007/s12325-022-02232-z 35844007 PMC9402774

[14] He H Liu Q Li N Guo L Gao F Bai L et al. Trends in the incidence and DALYs of schizophrenia at the global, regional and national levels: results from the Global Burden of Disease Study 2017. Epidemiol Psychiatr Sci. (2020) 29:e91. 10.1017/S2045796019000891 31928566 PMC7214712

[15] Cho SJ Kim J Kang YJ Lee SY Seo HY Park JE. Annual prevalence and incidence of schizophrenia and similar psychotic disorders in the Republic of Korea: A national health insurance data-based study. Psychiatry Investig. (2020) 17:61–70. 10.30773/pi.2019.0041 31995973 PMC6992854

[16] Chien IC Chou YJ Lin CH Bih SH Chou P Chang HJ. Prevalence and incidence of schizophrenia among national health insurance enrollees in Taiwan, 1996-2001. Psychiatry Clin Neurosci. (2004) 58:611–8. 10.1111/j.1440-1819.2004.01311.x 15601385

[17] Vermeulen J van Rooijen G Doedens P Numminen E van Tricht M de Haan L. Antipsychotic medication and long-term mortality risk in patients with schizophrenia: a systematic review and meta-analysis. Psychol Med. (2017) 47:2217–28. 10.1017/S0033291717000873 28397632

[18] Joukamaa M. Heliövaara M, Knekt P, Aromaa A, Raitasalo R, Lehtinen V. Schizophrenia, neuroleptic medication, and mortality. Br J Psychiatry. (2006) 188:122–7. 10.1192/bjp.188.2.122 16449697

[19] Marder SR Essock SM Miller AL Buchanan RW Casey DE Davis JM. Physical health monitoring of patients with schizophrenia. Am J Psychiatry. (2004) 161:1334–49. 10.1176/appi.ajp.161.8.1334 15285957

[20] Chang CK Chesney E Teng WN Hollandt S Pritchard M Shetty H. Life expectancy, mortality risks and cause of death in patients with serious mental illness in South East London: a comparison between 2008-2012 and 2013-2017. Psychol Med. (2023) 53:887–96. 10.1017/S0033291721002257 37132645 PMC9975985

[21] Björk Brämberg E Torgerson J Norman Kjellström A Welin P Rusner M. Access to primary and specialized somatic health care for persons with severe mental illness: a qualitative study of perceived barriers and facilitators in Swedish health care. BMC Fam Pract. (2018) 19:12. 10.1186/s12875-017-0687-0 29316894 PMC5759233

[22] Saha S Chant D McGrath J. A systematic review of mortality in schizophrenia. Arch Gen Psychiatry. (2007) 64:1123–31. 10.1001/archpsyc.64.10.1123 17909124

[23] Chan JKN Wong CSM Or PCF Chen EYH Chang WC. Risk of mortality and complications in patients with schizophrenia and diabetes mellitus: population-based cohort study. Br J Psychiatry. (2021) 219:375–82. 10.1192/bjp.2020.248 33407970

[24] Sudarshan Y Cheung BMY. Hypertension and psychosis. Postgrad Med J. (2023) 99:411–5. 10.1136/postgradmedj-2021-141386 37294717

[25] Westman J Eriksson SV Gissler M Hällgren J Prieto ML Bobo WV. Increased cardiovascular mortality in people with schizophrenia: a 24-year national register study. Epidemiol Psychiatr Sci. (2018) 27:519–27. 10.1017/S2045796017000166 28580898 PMC6137375

[26] Suetani S Honarparvar F Siskind D Hindley G Veronese N Vancampfort D. Increased rates of respiratory disease in schizophrenia: a systematic review and meta-analysis including 619,214 individuals with schizophrenia and 52,159,551 controls. Schizophr Res. (2021) 237:131–40. 10.1016/j.schres.2021.08.022 34521040

[27] Ali S Santomauro D Ferrari AJ Charlson F. Schizophrenia as a risk factor for cardiovascular and metabolic health outcomes: a comparative risk assessment. Epidemiol Psychiatr Sci. (2023) 32:e8. 10.1017/S2045796023000045 36756905 PMC9971851

[28] Nordentoft M Plana-Ripoll O Laursen TM. Cancer and schizophrenia. Curr Opin Psychiatry. (2021) 34:260–5. 10.1097/YCO.0000000000000697 33560020

[29] Charlson FJ Baxter AJ Dua T Degenhardt L Whiteford HA Vos T. Excess mortality from mental, neurological and substance use disorders in the Global Burden of Disease Study 2010. Epidemiol Psychiatr Sci. (2015) 24:121–40. 10.1017/S2045796014000687 25497332 PMC6998140

[30] Galderisi S De Hert M Del Prato S Fagiolini A Gorwood P Leucht S. Identification and management of cardiometabolic risk in subjects with schizophrenia spectrum disorders: a Delphi expert consensus study. Eur Psychiatry. (2021) 64:e7. 10.1192/j.eurpsy.2020.115 33413701 PMC8057390

[31] Peritogiannis V Ninou A Samakouri M. Mortality in schizophrenia- spectrum disorders: recent advances in understanding and management. Healthcare (Basel). (2022) 10:2366. 10.3390/healthcare10122366 36553890 PMC9777663

[32] De Hert M Vancampfort D Correll CU Mercken V Peuskens J Sweers K. Guidelines for screening and monitoring of cardiometabolic risk in schizophrenia: systematic evaluation. Br J Psychiatry. (2011) 199:99–105. 10.1192/bjp.bp.110.084665 21804146

[33] Busner J Targum SD. The clinical global impressions scale: applying a research tool in clinical practice. Psychiatry (Edgmont). (2007) 4:28–37. PMC2880930 20526405

[34] Health Promotion Administration. Ministry of Health and Welfare. Taiwan's Obesity Prevention and Management Strategy. 1st edn, 1, 55. Health Promotion Administration, Ministry of Health and Welfare (2018).

[35] Byrne BM. Structural Equation Modeling with AMOS: Basic Concepts, Applications, and Programming. New York: Routledge. (2009).

[36] Correll CU Solmi M Croatto G Schneider LK Rohani-Montez SC Fairley L. Mortality in people with schizophrenia: a systematic review and meta-analysis of relative risk and aggravating or attenuating factors. World Psychiatry. (2022) 21:248–71. 10.1002/wps.20994 35524619 PMC9077617

[37] Tsai KY Lee CC Chou YM Su CY Chou FH. The incidence and relative risk of stroke in patients with schizophrenia: a five-year follow-up study. Schizophr Res. (2012) 138:41–7. 10.1016/j.schres.2012.02.013 22386734

[38] Kapral MK Kurdyak P Casaubon LK Fang J Porter J Sheehan KA. Stroke care and case fatality in people with and without schizophrenia: a retrospective cohort study. BMJ Open. (2021) 11:e044766. 10.1136/bmjopen-2020-044766 34112641 PMC8194334

[39] Kelly DL McMahon RP Wehring HJ Liu F Mackowick KM Boggs DL. Cigarette smoking and mortality risk in people with schizophrenia. Schizophr Bull. (2011) 37:832–8. 10.1093/schbul/sbp152 20019128 PMC3122289

[40] Osborn DP Nazareth I King MB. Physical activity, dietary habits and Coronary Heart Disease risk factor knowledge amongst people with severe mental illness: a cross sectional comparative study in primary care. Soc Psychiatry Psychiatr Epidemiol. (2007) 42:787–93. 10.1007/s00127-007-0247-3 17721669

[41] Chen J Perera G Shetty H Broadbent M Xu Y Stewart R. Body mass index and mortality in patients with schizophrenia spectrum disorders: a cohort study in a South London catchment area. Gen Psychiatr. (2022) 35:e100819. 10.1136/gpsych-2022-100819 36447757 PMC9639123

[42] Hsu MC Ouyang WC. Subsequent dyslipidemia and factors associated with mortality in schizophrenia: a population-based study in Taiwan. Healthcare (Basel). (2021) 9:545. 10.3390/healthcare9050545 34067015 PMC8150361

[43] Cheng CS Chen WY Chang HM Pan CH Su SS Tsai SY. Unfavorable cancer mortality-to-incidence ratios in patients with schizophrenia: a nationwide cohort study in Taiwan, 2000-2019. Acta Psychiatr Scand. (2023) 148:347–58. 10.1111/acps.13604 37607118

[44] Ko YS Tsai HC Chi MH Su CC Lee IH Chen PS. Higher mortality and years of potential life lost of suicide in patients with schizophrenia. Psychiatry Res. (2018) 270:531–7. 10.1016/j.psychres.2018.09.038 30342411

[45] Mitchell AJ Vancampfort D De Herdt A Yu W De Hert M. Is the prevalence of metabolic syndrome and metabolic abnormalities increased in early schizophrenia? A comparative meta-analysis of first episode, untreated and treated patients. Schizophr Bull. (2013) 39:295–305. 10.1093/schbul/sbs082 22927670 PMC3576152

[46] Stubbs B Vancampfort D De Hert M Mitchell AJ. The prevalence and predictors of type two diabetes mellitus in people with schizophrenia: a systematic review and comparative meta-analysis. Acta Psychiatr Scand. (2015) 132:144–57. 10.1111/acps.12439 25943829

[47] Mukherjee S Schnur DB Reddy R. Family history of type 2 diabetes in schizophrenic patients. Lancet. (1989) 1:495. 10.1016/S0140-6736(89)91392-5 2563862

[48] Miron IC Baroană VC Popescu F Ionică F. Pharmacological mechanisms underlying the association of antipsychotics with metabolic disorders. Curr Health Sci J. (2014) 40:12–7. 24791199 10.12865/CHSJ.40.01.02 PMC4006340

[49] Brenner K Liu A Laplante DP Lupien S Pruessner JC Ciampi A. Cortisol response to a psychosocial stressor in schizophrenia: blunted, delayed, or normal? Psychoneuroendocrinology. (2009) 34:859–68. 10.1016/j.psyneuen.2009.01.002 19195793

[50] Onaolapo OJ Onaolapo AY. Nutrition, nutritional deficiencies, and schizophrenia: an association worthy of constant reassessment. World J Clin Cases. (2021) 9:8295–311. 10.12998/wjcc.v9.i28.8295 34754840 PMC8554424

[51] Andrews RC. Diabetes and schizophrenia: genes or zinc deficiency? Lancet. (1992) 340:1160. 10.1016/0140-6736(92)93186-Q 1359228

[52] Ratliff JC Palmese LB Reutenauer EL Liskov E Grilo CM Tek C. The effect of dietary and physical activity pattern on metabolic profile in individuals with schizophrenia: a cross-sectional study. Compr Psychiatry. (2012) 53:1028–33. 10.1016/j.comppsych.2012.02.003 22425530 PMC3380150

[53] Daumit GL Goldberg RW Anthony C Dickerson F Brown CH Kreyenbuhl J. Physical activity patterns in adults with severe mental illness. J Nerv Ment Dis. (2005) 193:641–6. 10.1097/01.nmd.0000180737.85895.60 16208158

[54] Suzuki Y Sugai T Fukui N Watanabe J Ono S Tsuneyama N. High prevalence of underweight and undernutrition in Japanese inpatients with schizophrenia. Psychiatry Clin Neurosci. (2014) 68:78–82. 10.1111/pcn.12082 23992354

[55] Dipasquale S Pariante CM Dazzan P Aguglia E McGuire P Mondelli V. The dietary pattern of patients with schizophrenia: a systematic review. J Psychiatr Res. (2013) 47:197–207. 10.1016/j.jpsychires.2012.10.005 23153955

[56] Nielsen RE Banner J Jensen SE. Cardiovascular disease in patients with severe mental illness. Nat Rev Cardiol. (2021) 18:136–45. 10.1038/s41569-020-00463-7 33128044

[57] Osimo EF Brugger SP de Marvao A Pillinger T Whitehurst T Statton B. Cardiac structure and function in schizophrenia: cardiac magnetic resonance imaging study. Br J Psychiatry. (2020) 217:450–7. 10.1192/bjp.2019.268 31915079 PMC7511899

[58] Korkmaz S Korkmaz H Özer Ö Atmaca M. Assessment of left ventricle systolic and diastolic functions in schizophrenia patients. Psychiatry Res. (2016) 240:348–51. 10.1016/j.psychres.2016.04.025 27138830

[59] Kilbourne AM Morden NE Austin K Ilgen M McCarthy JF Dalack G. Excess heart-disease-related mortality in a national study of patients with mental disorders: identifying modifiable risk factors. Gen Hosp Psychiatry. (2009) 31:555–63. 10.1016/j.genhosppsych.2009.07.008 19892214 PMC4033835

[60] Liao CH Chang CS Chang SN Muo CH Lane HY Sung FC. The association of peptic ulcer and schizophrenia: a population-based study. J Psychosom Res. (2014) 77:541–6. 10.1016/j.jpsychores.2014.08.005 25199406

[61] Kasap E Ayer A Bozoglan H Ozen C Eslek I Yüceyar H. Schizophrenia and gastroesophageal reflux symptoms. Indian J Psychiatry. (2015) 57:73–7. 10.4103/0019-5545.148529 25657460 PMC4314920

[62] Nielsen J Meyer JM. Risk factors for ileus in patients with schizophrenia. Schizophr Bull. (2012) 38:592–8. 10.1093/schbul/sbq137 21112965 PMC3329981

[63] Huang TL. Decreased serum albumin levels in Taiwanese patients with schizophrenia. Psychiatry Clin Neurosci. (2002) 56:627–30. 10.1046/j.1440-1819.2002.01066.x 12485305

[64] Moramarco S Morciano L Morucci L Messinese M Gualtieri P Carestia M. Epidemiology of hypoalbuminemia in hospitalized patients: a clinical matter or an emerging public health problem? Nutrients. (2020) 12:3656. 10.3390/nu12123656 33261019 PMC7760225

[65] Feldman JG Burns DN Gange SJ Bacchetti P Cohen M Anastos K. Serum albumin as a predictor of survival in HIV-infected women in the Women's Intergency HIV study. AIDS. (2000) 14:863–70. 10.1097/00002030-200005050-00013 10839595

[66] Don BR Kaysen G. Serum albumin: relationship to inflammation and nutrition. Semin Dial. (2004) 17:432–7. 10.1111/j.0894-0959.2004.17603.x 15660573

[67] Korkmaz S Yildiz S Korucu T Gundogan B Sunbul ZE Korkmaz H. Frequency of anemia in chronic psychiatry patients. Neuropsychiatr Dis Treat. (2015) 11:2737–41. 10.2147/NDT.S91581 26543367 PMC4622486

[68] Polcwiartek C Loewenstein D Friedman DJ Johansson KG Graff C Sørensen PL. Clinical heart failure among patients with and without severe mental illness and the association with long-term outcomes. Circ Heart Fail. (2021) 14:e008364. 10.1161/CIRCHEARTFAILURE.121.008364 34587762

[69] Adams SB. Recognition and management of ileus. Vet Clin North Am Equine Pract. (1988) 4:91–104. 10.1016/S0749-0739(17)30652-1 3289699

[70] Grant RK Brindle WM Donnelly MC McConville PM Stroud TG Bandieri L. Gastrointestinal and liver disease in patients with schizophrenia: a narrative review. World J Gastroenterol. (2022) 28:5515–29. 10.3748/wjg.v28.i38.5515 36304087 PMC9594005

[71] Yang Y Wan C Li H Zhu H La Y Xi Z. Altered levels of acute phase proteins in the plasma of patients with schizophrenia. Anal Chem. (2006) 78:3571–6. 10.1021/ac051916x 16737209

[72] Wan C La Y Zhu H Yang Y Jiang L Chen Y. Abnormal changes of plasma acute phase proteins in schizophrenia and the relation between schizophrenia and haptoglobin (Hp) gene. Amino Acids. (2007) 32:101–8. 10.1007/s00726-005-0292-8 16897611

[73] Lai KY Wu TH Liu CS Lin CH Lin CC Lai MM. Body mass index and albumin levels are prognostic factors for long-term survival in elders with limited performance status. Aging (Albany NY). (2020) 12:1104–13. 10.18632/aging.102642 31945744 PMC7053589

[74] Kamruzzaman M. Is BMI associated with anemia and hemoglobin level of women and children in Bangladesh: a study with multiple statistical approaches. PLoS ONE. (2021) 16:e0259116. 10.1371/journal.pone.0259116 34710186 PMC8553127

[75] Qian WL Xu R Shi R Li Y Guo YK Fang H. et al. The worsening effect of anemia on left ventricular function and global strain in type 2 diabetes mellitus patients: a 30 T CMR feature tracking study. Cardiovasc Diabetol. (2023) 22:15. 10.1186/s12933-023-01745-3 36694151 PMC9875473

[76] Siddiqui SW Ashok T Patni N Fatima M Lamis A Anne KK. Anemia and heart failure: a narrative review. Cureus. (2022) 14:e27167. 10.7759/cureus.27167 36017290 PMC9393312

[77] Oh GC Cho HJ. Blood pressure and heart failure. Clin Hypertens. (2020) 26:1. 10.1186/s40885-019-0132-x 31908841 PMC6939331

